# Tubarial Salivary Glands in Sjogren Syndrome: Are They Just a Potential Missing Link With No Broader Implications?

**DOI:** 10.3389/fimmu.2021.684490

**Published:** 2021-06-29

**Authors:** Alexios-Fotios A. Mentis, George P. Chrousos

**Affiliations:** ^1^ University Research Institute of Maternal and Child Health and Precision Medicine, and UNESCO Chair on Adolescent Health Care, Medical School, National and Kapodistrian University of Athens, Athens, Greece; ^2^ US National Academy of Medicine, Washington, DC, United States

**Keywords:** Sjogren’s syndrome, autoimmunity, salivary glands, discovery, tubarial glands

## Introduction

Sjogren syndrome (SS) is historically the prototype autoimmune disease of the moisture-producing salivary and lacrimal glands, leading respectively to dry mouth and eyes, and represents an exemplar autoimmune disease for the molecular immunologist (1). SS is also a systemic disease with manifestations from several organs, notably including the lungs, the kidneys, the liver, and the vascular system [for a description of the full clinical spectrum of SS, see (2)]. Therefore, treatment for SS should include addressing both local symptoms (i.e., xerosis), but also the underlying immunological aetiopathogenic mechanisms (3).

As a model autoimmune disease, SS has been the basis of a four decades-spanning scientific journey (4); during these forty years, many exciting findings have appeared conveying broader implications for autoimmune diseases and causing shifts in SS clinical management. Indicatively, *a)* the preliminary criteria for SS (5) have been thoroughly re-assessed, revised, and updated (6–8); *b)* the risk factors for lymphoma development, in particular Non-Hodgkin lymphoma in SS patients, has now been well-established (9) [whose development can be predicted with very high accuracy using appropriate predictive risk scores (10)]; hence, nowadays we know that lymphoma causes 20% of deaths in patients with SS (11); and, *c)* SS is an area of intensive recent clinical trials in the quest of effective treatment for a disease affecting 2 to 4 million people only in US (12). Of note, there is a recent, resurgent interest in SS in light of efforts to use immunomodulatory drugs for its control, whose efficacy though has not been proven in this condition as yet (13); however, hopes for improving symptoms of dry mouth and eyes with such agents remain high.

## Tubarial Glands: A Recent (RE)Discovery Applied To Sjogren Syndrome?

Intriguingly, a recent publication could provide unexpected clues to the SS arena even if it stems from the field of radiotherapy (14). According to the study’s findings, new bilateral salivary glands, called *tubarial glands*, −which are apparent even at the macroscopic level−, were recently described in patients with cancer. This observation was made possible using prostate-specific membrane antigen ligands (PSMA PET/CT) in a recently employed nuclear medicine approach that bears high diagnostic accuracy for salivary glands (15); actually, the finding was confirmed histologically in cadavers (14). Thus, tubarial glands are described as being located between the throat and the nasal cavity, notably *torus tubarius*, from which they derive their name. In addition, radiotherapy including the pharyngeal area of these glands led to xerostomia and dysphagia, suggesting, first, a major functional role for these recently described organs, and, second, a potential for averting these symptoms by avoiding radiation of this region (14).

While fully acknowledging the differences in focus of the consideration below, could these recently identified salivary glands have significant implications for SS? We contend that significance in light of their location and possible functionality and, hence, suggest that it would be worth investigating whether these glands are compromised in SS patients, not least in the subcategory of those with diagnosed SS and a dry rhinopharynx. Were this the case, it could provide a major anatomical clue to a SS subcategory and could be the impetus for future studies in the field. To this end, a multi-center PSMA PET/CT study of SS patients with or without a dry rhinopharynx could shed light into tubarial glands’ role in SS. If it were possible to anatomically access these glands and to conduct biopsies, one could confirm whether they are involved in SS or not. In the former case, SS classification and diagnostic criteria might be reformed (16).

Nonetheless, several critiques (expressed as letters to the Editor) have been expressed about this discovery. The concerns expressed refer to *a)* the small patient population and the confirmatory data only obtained in two cadavers; *b)* the male predominance in the study’s participants with prostate or urethral gland cancer and only a single woman, thus indicating a lack of diversity of the population studied (17); *c)* whether these glands are true salivary glands in the sense that they contribute to saliva’s production, granted that they lack amylase (18); and, *d)* whether these glands are truly novel and not a redescription of findings reported by 19^th^ anatomists (19). Moreover, concerns have been raised on the imaging techniques that were applied for the identification of tumor growths in a specific patient population rather than in the general population.

Hence, in light of the lack of consensus and current uncertainties on the tubarial glands, the opinion we here-in propose would require first the above work to be widened to include *a)* larger populations, *b)* other confirmatory imagining methods, *c)* further studies in female patients, and *d)* more extensive histological and immunohistochemical analyses, including comparison with findings described in major and minor labial salivary glands of patients with SS.

## Tubarial glands: A redisocvery with broader implications?

We also contend that regardless of whether the above investigation is a mere academic exercise or one potentially ending in changing SS-related clinical practice, the implications may raise a broader scientific question, i.e., should these observations be treated as a discovery or re-discovery? Similarly, other recent discoveries have been made regarding anatomical descriptions and sites that were presented as novel, namely *a)* the meningeal lymphatic vessels (20); *b)* the interstitium as a fluid-filling space between cells (21); *c)* the mesentery as a complete organ instead of fragmented parts (22); *d)* the discovery of arterial and venous capillaries in long bones (23); *e)* the presence of *atavistic* limb muscles in human embryos (24); and, *f)* the increase in the incidence of the sesamoid bone fabella over the last 150 years (25). Criticisms on the novelty of these findings have been expressed and collectively discussed (26). Undeniably, having missed key macro- and micro-scopic anatomical organs, such as the above, could be a major issue of concern in an otherwise high-throughput, single-cell molecular pathology era (27). Perhaps, more important may be to *invest on mining hidden pearls that can be crucial in advancing biomedical research* [as we advocated previously (28)], especially in the current era that focuses on *ad nauseam* confirmations of previous findings using new analytical tools (29).

## Conclusion

Although our opinion article can be regarded as highly speculative because of the lack of general acceptance of the unequivocality of the findings on the tubarial glands, we wish to suggest that the tubarial glands may be implicated in SS. This may explain why some patients with SS present with upper airway dryness, ultimately leading to improve SS classification and diagnostic criteria. It is, thus, high time for the biomedical research community to stop neglecting potentially important anatomical research questions, whose understanding is pivotal in the advancement of biomedical research.

## Author Contributions

A-FM and GC conceived this opinion article. A-FM provided the initial content and draft for the manuscript, and GC improved the manuscript content with further literature and critical points. All authors contributed to the article and approved the submitted version.

## Conflict of Interest

The authors declare that the research was conducted in the absence of any commercial or financial relationships that could be construed as a potential conflict of interest.
